# Supplementary material for: Gender with marital status, cultural differences, and vulnerability to hypertension: Findings from the national survey for noncommunicable disease risk factors and mental health using WHO STEPS in Bhutan
Source: PLoS One. 2021 Aug 31;16(8):e0256811. doi: 10.1371/journal.pone.0256811 (PMC8407566; doi:10.1371/journal.pone.0256811)
Supplement: S1 File — (PDF) [file pone.0256811.s001.pdf]

S1 File. Sample size and survey procedures quoted from the original report of the World Health Organization and Ministry of Health in Bhutan (National survey for noncommunicable disease risk factors and mental health using WHO STEPS approach in Bhutan – 2014).

“The Sample size estimate of the number of households to be surveyed with 95% confidence was calculated using the following formula and assumptions.

$$n = \frac{Z^2{}_{1-\alpha} P(1-P)}{d^2}$$

Where:

Z = level of confidence measure; this represents the number of standard errors away from the mean and describes the uncertainty in the sample mean or prevalence as an estimate of the population mean (normal deviation if alpha equals 0.05, then Z = 1.96 for 95% confidence level). P = baseline level of the indicators. This is the estimated proportion of one of the indicators related to the risk factors currently being measured. The prevalence of overweight and obesity was 52.8% from the last STEPS survey carried out in Thimphu which was the closest value to 50%. d = margin of error. The expected half width of the confidence interval was taken as 0.05 for this study

$$n = \frac{1.96 * 1.96\ \{0.528(1-0.528)\}}{0.05 * 0.05}$$

$$n = 382.9552$$

Four domains were chosen based on men and women and two age groups: younger (18–39 years) and older (40–69 years), providing four age/sex estimates. Taking into account the number of domains and ensuring enough representation by either age-sex groups or urban-rural groups in men and women, and with a design effect of 1.5 to address the issue of cluster sampling, the expected sample size was as follows:

$$\boldsymbol{n} = \mathbf{382.9552 * 1.5 * 4 = 2297.7316}$$

Assuming an expected 80% response rate, the final required sample size was 2912.

$$\boldsymbol{n} = \mathbf{2297.7316/0.8 = 2872.1646\sim} \text{ (rounded to 2912 for logistical ease)}$$

Out of the targeted 2912 respondents, 2822 (96.9%) participated in STEP I (behavioral measurement), and 2816 (96.7%) participated in STEP 2 (physical measurements). For STEP 3 (biochemical measurement), the response rate for the fasting blood glucose test, excluding non-fasting respondents, was 93.5% (2724 respondents), that for total cholesterol levels was 94.8% (2761 respondents), and urine collection for salt estimation was 89.9% (2618 respondents).”
