# Supplementary figures and images for: Gender with marital status, cultural differences, and vulnerability to hypertension: Findings from the national survey for noncommunicable disease risk factors and mental health using WHO STEPS in Bhutan
Source: PLoS One. 2021 Aug 31;16(8):e0256811. doi: 10.1371/journal.pone.0256811 (PMC8407566; doi:10.1371/journal.pone.0256811)

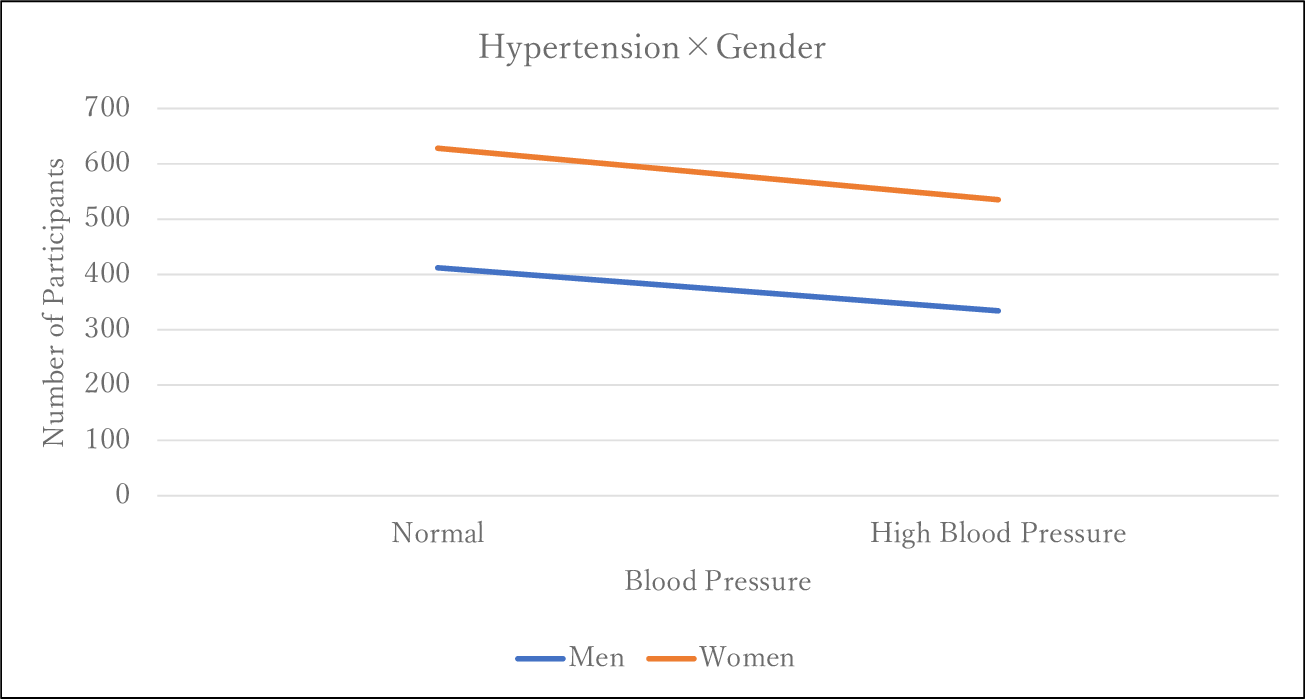

Supplement: S1 Fig — (TIF) [file pone.0256811.s002.tif]

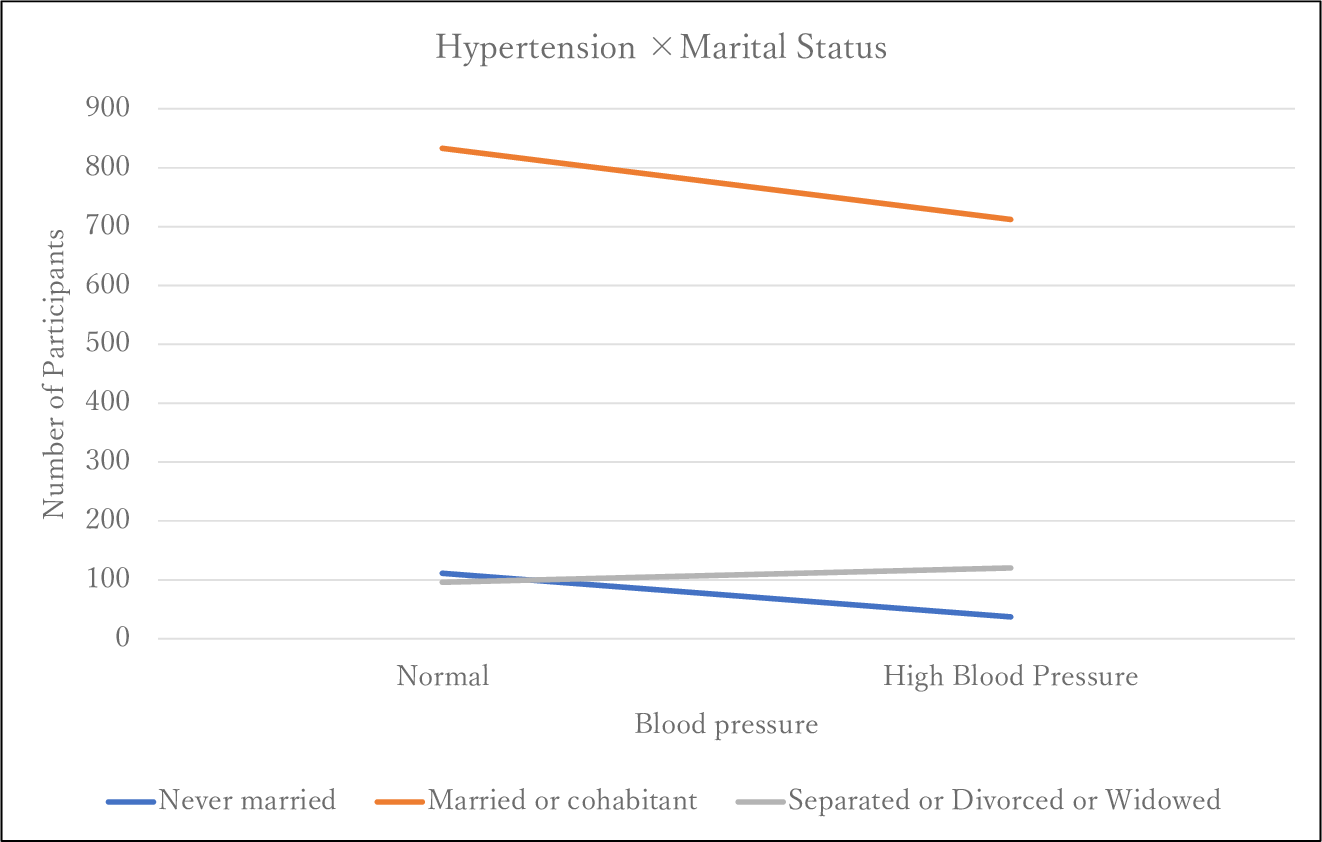

Supplement: S2 Fig — (TIF) [file pone.0256811.s003.tif]

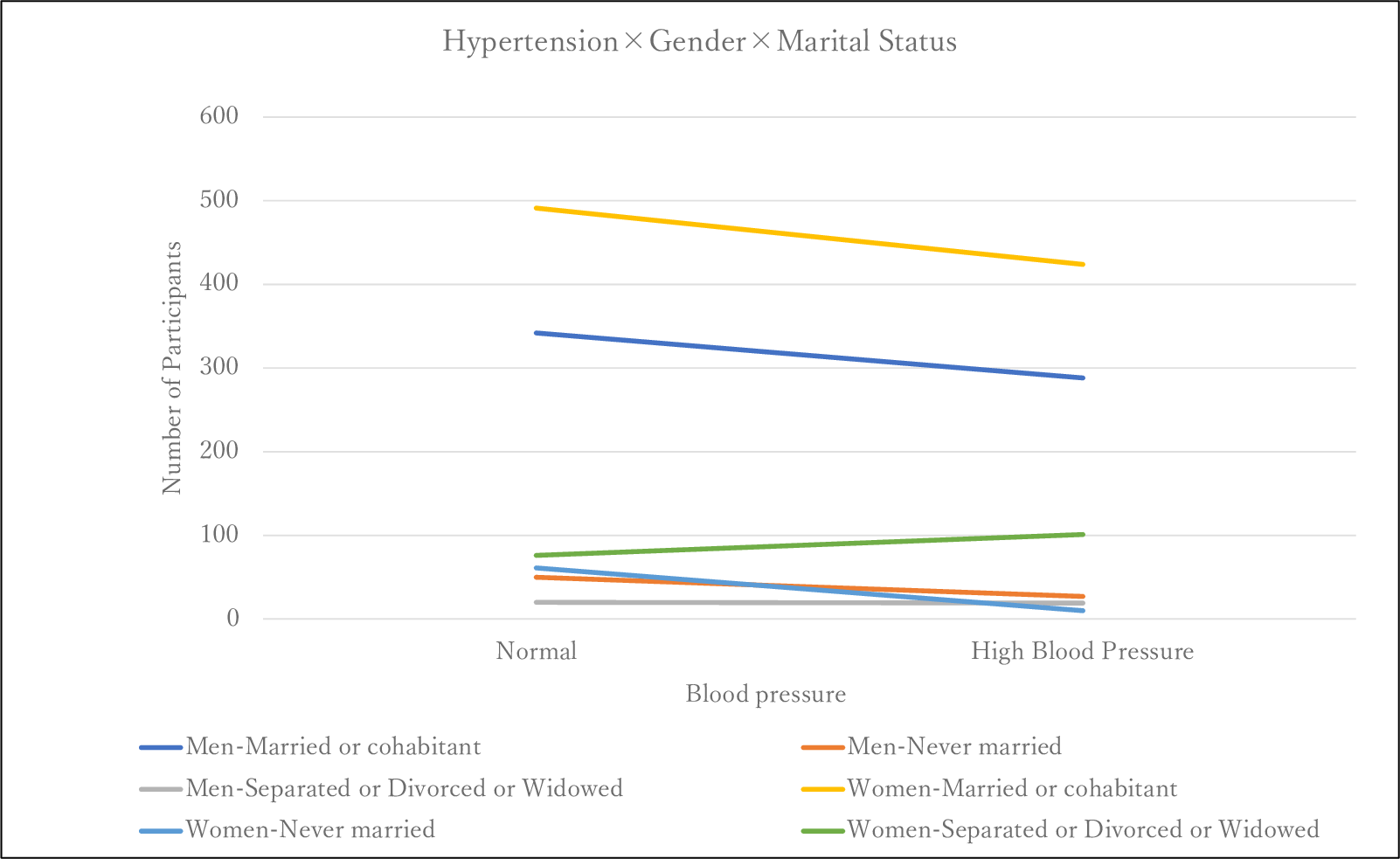

Supplement: S3 Fig — (TIF) [file pone.0256811.s004.tif]
