# Supplementary material for: Gender with marital status, cultural differences, and vulnerability to hypertension: Findings from the national survey for noncommunicable disease risk factors and mental health using WHO STEPS in Bhutan
Source: PLoS One. 2021 Aug 31;16(8):e0256811. doi: 10.1371/journal.pone.0256811 (PMC8407566; doi:10.1371/journal.pone.0256811)
Supplement: S1 Table — (DOCX) [file pone.0256811.s005.docx]

**S1** **Table. Multivariable Logistic Regression Analysis for Hypertension with Sociocultural, Lifestyle, Biomedical, and Mental Health Variables: without considering interaction effects (n = 1,909)**

|  | | | | | | | | | | | | | | | | | | |
| --- | --- | --- | --- | --- | --- | --- | --- | --- | --- | --- | --- | --- | --- | --- | --- | --- | --- | --- |
|  |  | Non-weighted | | | | | | | | Weighted | | | | | | | |  |
| n=1909 |  | AOR^1)^ | 95%CI | | | | | | *p*-value | AOR^2)^ | 95%CI | | | | | | *p-*value |  |
| Gender | Men | Ref | ( |  | - |  | ) |  | | Ref | ( |  | - |  | ) |  | |  |
|  | Women | 1.08 | ( | 0.85 | - | 1.38 | ) | 0.511 | | 1.14 | ( | 1.11 | - | 1.17 | ) | <0.001 | |  |
| Marital Status | Married or cohabitant | Ref | ( |  | - |  | ) |  | | Ref | ( |  | - |  | ) |  | |  |
|  | Never married | 0.90 | ( | 0.56 | - | 1.43 | ) | 0.641 | | 0.80 | ( | 0.76 | - | 0.84 | ) | <0.001 | |  |
|  | Separated or Divorced or Widow | 0.97 | ( | 0.55 | - | 1.69 | ) | 0.906 | | 0.69 | ( | 0.65 | - | 0.74 | ) | <0.001 | |  |
| Age | 18-29 years | Ref | ( |  | - |  | ) |  | | Ref | ( |  | - |  | ) |  | |  |
|  | 30-39 years | 2.13 | ( | 1.56 | - | 2.92 | ) | <0.001 | | 2.47 | ( | 2.39 | - | 2.56 | ) | <0.001 | |  |
|  | 40-49 years | 3.52 | ( | 2.52 | - | 4.91 | ) | <0.001 | | 3.41 | ( | 3.29 | - | 3.55 | ) | <0.001 | |  |
|  | 50-59 years | 4.70 | ( | 3.22 | - | 6.86 | ) | <0.001 | | 5.45 | ( | 5.21 | - | 5.69 | ) | <0.001 | |  |
|  | 60-69 years | 7.46 | ( | 4.73 | - | 11.76 | ) | <0.001 | | 8.35 | ( | 7.90 | - | 8.82 | ) | <0.001 | |  |
| Education-years | No formal education | Ref | ( |  | - |  | ) |  | | Ref | ( |  | - |  | ) |  | |  |
|  | 1-10 years | 0.92 | ( | 0.71 | - | 1.18 | ) | 0.489 | | 0.94 | ( | 0.92 | - | 0.97 | ) | <0.001 | |  |
|  | 11-12 years | 0.59 | ( | 0.31 | - | 1.12 | ) | 0.105 | | 0.65 | ( | 0.61 | - | 0.69 | ) | <0.001 | |  |
|  | More than 12 years | 0.96 | ( | 0.47 | - | 1.95 | ) | 0.911 | | 0.95 | ( | 0.88 | - | 1.02 | ) | 0.164 | |  |
| Working Status | Employee | Ref | ( |  | - |  | ) |  | | Ref | ( |  | - |  | ) |  | |  |
|  | Self-employed | 0.96 | ( | 0.69 | - | 1.35 | ) | 0.823 | | 1.07 | ( | 1.03 | - | 1.11 | ) | <0.001 | |  |
|  | Non-working | 0.88 | ( | 0.61 | - | 1.25 | ) | 0.465 | | 0.92 | ( | 0.88 | - | 0.96 | ) | <0.001 | |  |
| Residential area | Rural | Ref | ( |  | - |  | ) |  | | Ref | ( |  | - |  | ) |  | |  |
|  | Urban | 0.98 | ( | 0.75 | - | 1.28 | ) | 0.869 | | 1.12 | ( | 1.09 | - | 1.16 | ) | <0.001 | |  |
| Income | Nu.0-9,000 | Ref | ( |  | - |  | ) |  | | Ref | ( |  | - |  | ) |  | |  |
|  | Nu.9,001-30,000 | 1.12 | ( | 0.85 | - | 1.47 | ) | 0.415 | | 0.90 | ( | 0.88 | - | 0.93 | ) | <0.001 | |  |
|  | Nu.30,001-60,000 | 1.08 | ( | 0.78 | - | 1.48 | ) | 0.652 | | 0.73 | ( | 0.71 | - | 0.76 | ) | <0.001 | |  |
|  | Nu.60,001- | 1.17 | ( | 0.84 | - | 1.62 | ) | 0.364 | | 1.03 | ( | 0.99 | - | 1.07 | ) | 0.160 | |  |
| Survey language | Dzongkha | Ref | ( |  | - |  | ) |  | | Ref | ( |  | - |  | ) |  | |  |
|  | Tshanglakha | 0.00 | ( | 1.29 | - | 2.20 | ) | 1.687 | | 1.21 | ( | 1.18 | - | 1.25 | ) | <0.001 | |  |
|  | Lhotshamkha | 0.03 | ( | 1.04 | - | 1.76 | ) | 1.348 | | 1.08 | ( | 1.05 | - | 1.11 | ) | <0.001 | |  |
|  | English | 0.05 | ( | 0.10 | - | 0.99 | ) | 0.308 | | 0.28 | ( | 0.25 | - | 0.32 | ) | <0.001 | |  |
| Tobacco use | Never use | Ref | ( |  | - |  | ) |  | | Ref | ( |  | - |  | ) |  | |  |
|  | Currently use | 0.04 | ( | 0.58 | - | 0.99 | ) | 0.753 | | 0.82 | ( | 0.80 | - | 0.84 | ) | <0.001 | |  |
| Alcohol consumption | Never drink | Ref | ( |  | - |  | ) |  | | Ref | ( |  | - |  | ) |  | |  |
|  | Light or moderate drinking | 1.25 | ( | 0.98 | - | 1.59 | ) | 0.075 | | 1.22 | ( | 1.19 | - | 1.26 | ) | <0.001 | |  |
|  | Heavy drinking | 1.55 | ( | 1.18 | - | 2.04 | ) | 0.001 | | 1.33 | ( | 1.29 | - | 1.37 | ) | <0.001 | |  |
| Fruit and vegetable consumption | More than 5 serves per day | Ref | ( |  | - |  | ) |  | | Ref | ( |  | - |  | ) |  | |  |
|  | 5 or fewer serves per day | 1.09 | ( | 0.87 | - | 1.37 | ) | 0.455 | | 0.96 | ( | 0.94 | - | 0.99 | ) | 0.003 | |  |
| Physical Activity | 150 min or more per week | Ref | ( |  | - |  | ) |  | | Ref | ( |  | - |  | ) |  | |  |
|  | Less than 150 mins per week | 0.83 | ( | 0.56 | - | 1.23 | ) | 0.352 | | 0.85 | ( | 0.81 | - | 0.90 | ) | <0.001 | |  |
| Salt intake | Less than 5 g per day | Ref | ( |  | - |  | ) |  | | Ref | ( |  | - |  | ) |  | |  |
|  | 5 g or more per day | 1.45 | ( | 0.57 | - | 3.69 | ) | 0.440 | | 1.41 | ( | 1.24 | - | 1.60 | ) | <0.001 | |  |
| Blood glucose | Normal | Ref | ( |  | - |  | ) |  | | Ref | ( |  | - |  | ) |  | |  |
|  | Abnormal | 2.15 | ( | 1.45 | - | 3.19 | ) | <0.001 | | 2.25 | ( | 2.15 | - | 2.36 | ) | <0.001 | |  |
| Total cholesterol | Less than 240 mg/dl | Ref | ( |  | - |  | ) |  | | Ref | ( |  | - |  | ) |  | |  |
|  | 240 mg/dl or higher | 1.33 | ( | 0.65 | - | 2.72 | ) | 0.440 | | 1.68 | ( | 1.54 | - | 1.84 | ) | <0.001 | |  |
| BMI | <18.5 | Ref | ( |  | - |  | ) |  | | Ref | ( |  | - |  | ) |  | |  |
|  | 18.5 ≤ BMI < 25.0 | 1.54 | ( | 0.85 | - | 2.79 | ) | 0.152 | | 1.77 | ( | 1.66 | - | 1.89 | ) | <0.001 | |  |
|  | 25.0 ≤ BMI < 30.0 | 2.65 | ( | 1.44 | - | 4.87 | ) | 0.002 | | 2.96 | ( | 2.76 | - | 3.17 | ) | <0.001 | |  |
|  | ≥30.0 | 3.64 | ( | 1.84 | - | 7.22 | ) | <0.001 | | 4.25 | ( | 3.93 | - | 4.60 | ) | <0.001 | |  |
| Family history of hypertension | Negative | Ref | ( |  | - |  | ) |  | | Ref | ( |  | - |  | ) |  | |  |
|  | Positive | 1.34 | ( | 1.08 | - | 1.67 | ) | 0.009 | | 1.12 | ( | 1.09 | - | 1.15 | ) | <0.001 | |  |
| Cardiovascular disease | Negative | Ref | ( |  | - |  | ) |  | | Ref | ( |  | - |  | ) |  | |  |
|  | Positive | 0.86 | ( | 0.29 | - | 2.58 | ) | 0.792 | | 0.59 | ( | 0.51 | - | 0.68 | ) | <0.001 | |  |
| Considering Suicide | Negative | Ref | ( |  | - |  | ) |  | | Ref | ( |  | - |  | ) |  | |  |
|  | Positive | 0.77 | ( | 0.37 | - | 1.61 | ) | 0.495 | | 1.21 | ( | 1.12 | - | 1.32 | ) | <0.001 | |  |
| 1) Adjusted with all explanatory variables | | | | | | | | | | | | | | | | | |  |
| 2) Weight adjusted and adjusted with all explanatory variables | | | | | | | | | | | | | | | | | |  |
