# Supplementary material for: Gender with marital status, cultural differences, and vulnerability to hypertension: Findings from the national survey for noncommunicable disease risk factors and mental health using WHO STEPS in Bhutan
Source: PLoS One. 2021 Aug 31;16(8):e0256811. doi: 10.1371/journal.pone.0256811 (PMC8407566; doi:10.1371/journal.pone.0256811)
Supplement: S2 Table — (DOCX) [file pone.0256811.s006.docx]

**S2 Table. Multivariable Logistic Regression Analysis for Hypertension with Sociocultural, Lifestyle, Biomedical, and Mental Health Variables: including interaction effects (n = 1,909)**

|  |  | Non-weighted | | | | | | | Weighted | | | | | | |
| --- | --- | --- | --- | --- | --- | --- | --- | --- | --- | --- | --- | --- | --- | --- | --- |
| n=1909 |  | AOR^1)^ | 95%CI | | | | | *p*-value | AOR^2)^ | 95%CI | | | | | *p-*value |
| Gender | Men | Ref | ( |  | - |  | ) |  | Ref | ( |  | - |  | ) |  |
|  | Women | 0.26 | ( | 0.11 | - | 0.62 | ) | 0.003 | 0.22 | ( | 0.20 | - | 0.25 | ) | <0.001 |
| Marital Status | Married or cohabitant | Ref | ( |  | - |  | ) |  | Ref | ( |  | - |  | ) |  |
|  | Never married | 0.46 | ( | 0.26 | - | 0.84 | ) | 0.011 | 0.51 | ( | 0.49 | - | 0.54 | ) | <0.001 |
|  | Separated or Divorced or Widow | 0.37 | ( | 0.15 | - | 0.90 | ) | 0.028 | 0.33 | ( | 0.30 | - | 0.37 | ) | <0.001 |
| Interaction Effects | marital‎_cat3v * sexv |  | ( |  | - |  | ) | 0.003 | Ref | ( |  | - |  | ) |  |
|  | marital‎_cat3v(1) by sexv(1) | 4.61 | ( | 1.83 | - | 11.57 | ) | 0.001 | 5.69 | ( | 5.08 | - | 6.37 | ) | <0.001 |
|  | marital‎_cat3v(2) by sexv(1) | 6.57 | ( | 2.05 | - | 21.04 | ) | 0.002 | 8.14 | ( | 7.04 | - | 9.42 | ) | <0.001 |
| Age | 18-29 years | Ref | ( |  | - |  | ) |  | Ref | ( |  | - |  | ) |  |
|  | 30-39 years | 2.15 | ( | 1.57 | - | 2.96 | ) | <0.001 | 2.53 | ( | 2.44 | - | 2.61 | ) | <0.001 |
|  | 40-49 years | 3.64 | ( | 2.60 | - | 5.09 | ) | <0.001 | 3.50 | ( | 3.37 | - | 3.64 | ) | <0.001 |
|  | 50-59 years | 4.90 | ( | 3.35 | - | 7.17 | ) | <0.001 | 5.71 | ( | 5.46 | - | 5.97 | ) | <0.001 |
|  | 60-69 years | 7.79 | ( | 4.93 | - | 12.33 | ) | <0.001 | 8.78 | ( | 8.30 | - | 9.28 | ) | <0.001 |
| Education-years | No formal education | Ref | ( |  | - |  | ) |  | Ref | ( |  | - |  | ) |  |
|  | 1-10 years | 0.93 | ( | 0.73 | - | 1.20 | ) | 0.591 | 0.96 | ( | 0.94 | - | 0.99 | ) | 0.010 |
|  | 11-12 years | 0.57 | ( | 0.30 | - | 1.08 | ) | 0.086 | 0.64 | ( | 0.60 | - | 0.68 | ) | <0.001 |
|  | More than 12 years | 0.96 | ( | 0.47 | - | 1.96 | ) | 0.904 | 0.95 | ( | 0.88 | - | 1.03 | ) | 0.239 |
| Working Status | Employee | Ref | ( |  | - |  | ) |  | Ref | ( |  | - |  | ) |  |
|  | Self-employed | 0.92 | ( | 0.66 | - | 1.29 | ) | 0.644 | 1.02 | ( | 0.98 | - | 1.06 | ) | 0.331 |
|  | Non-working | 0.84 | ( | 0.59 | - | 1.21 | ) | 0.347 | 0.89 | ( | 0.85 | - | 0.92 | ) | <0.001 |
| Residential area | Rural | Ref | ( |  | - |  | ) |  | Ref | ( |  | - |  | ) |  |
|  | Urban | 0.97 | ( | 0.74 | - | 1.26 | ) | 0.797 | 1.10 | ( | 1.07 | - | 1.14 | ) | <0.001 |
| Income | Nu.0-9,000 | Ref | ( |  | - |  | ) |  | Ref | ( |  | - |  | ) |  |
|  | Nu.9,001-30,000 | 1.12 | ( | 0.85 | - | 1.47 | ) | 0.419 | 0.91 | ( | 0.88 | - | 0.94 | ) | <0.001 |
|  | Nu.30,001-60,000 | 1.07 | ( | 0.78 | - | 1.47 | ) | 0.683 | 0.73 | ( | 0.70 | - | 0.75 | ) | <0.001 |
|  | Nu.60,001- | 1.19 | ( | 0.85 | - | 1.66 | ) | 0.315 | 1.06 | ( | 1.02 | - | 1.10 | ) | 0.007 |
| Survey language | Dzongkha | Ref | ( |  | - |  | ) |  | Ref | ( |  | - |  | ) |  |
|  | Tshanglakha | 1.71 | ( | 1.31 | - | 2.23 | ) | <0.001 | 1.23 | ( | 1.19 | - | 1.27 | ) | <0.001 |
|  | Lhotshamkha | 1.36 | ( | 1.04 | - | 1.77 | ) | 0.024 | 1.08 | ( | 1.05 | - | 1.11 | ) | <0.001 |
|  | English | 0.30 | ( | 0.09 | - | 0.97 | ) | 0.044 | 0.28 | ( | 0.25 | - | 0.32 | ) | <0.001 |
| Tobacco use | Never use | Ref | ( |  | - |  | ) |  | Ref | ( |  | - |  | ) |  |
|  | Currently use | 0.75 | ( | 0.57 | - | 0.98 | ) | 0.033 | 0.81 | ( | 0.79 | - | 0.84 | ) | <0.001 |
| Alcohol consumption | Never drink | Ref | ( |  | - |  | ) |  | Ref | ( |  | - |  | ) |  |
|  | Light or moderate drinking | 1.26 | ( | 0.99 | - | 1.60 | ) | 0.066 | 1.23 | ( | 1.19 | - | 1.26 | ) | <0.001 |
|  | Heavy drinking | 1.54 | ( | 1.17 | - | 2.02 | ) | 0.002 | 1.31 | ( | 1.27 | - | 1.35 | ) | <0.001 |
| Fruit and vegetable consumption | More than 5 serves per day | Ref | ( |  | - |  | ) |  | Ref | ( |  | - |  | ) |  |
|  | 5 or fewer serves per day | 1.09 | ( | 0.87 | - | 1.38 | ) | 0.445 | 0.97 | ( | 0.94 | - | 0.99 | ) | 0.008 |
| Physical Activity | 150 min or more per week | Ref | ( |  | - |  | ) |  | Ref | ( |  | - |  | ) |  |
|  | Less than 150 min per week | 0.83 | ( | 0.56 | - | 1.22 | ) | 0.336 | 0.84 | ( | 0.80 | - | 0.88 | ) | <0.001 |
| Salt intake | Less than 5 g per day | Ref | ( |  | - |  | ) |  | Ref | ( |  | - |  | ) |  |
|  | 5 g ore more per day | 1.42 | ( | 0.55 | - | 3.61 | ) | 0.468 | 1.42 | ( | 1.25 | - | 1.62 | ) | <0.001 |
| Blood glucose | Normal | Ref | ( |  | - |  | ) |  | Ref | ( |  | - |  | ) |  |
|  | Abnormal | 2.13 | ( | 1.43 | - | 3.17 | ) | <0.001 | 2.27 | ( | 2.17 | - | 2.38 | ) | <0.001 |
| Total cholesterol | Less than 240 mg/dl | Ref | ( |  | - |  | ) |  | Ref | ( |  | - |  | ) |  |
|  | 240 mg/dl or more | 1.32 | ( | 0.64 | - | 2.70 | ) | 0.455 | 1.66 | ( | 1.52 | - | 1.81 | ) | <0.001 |
| BMI | <18.5 | Ref | ( |  | - |  | ) |  | Ref | ( |  | - |  | ) |  |
|  | 18.5 ≤ BMI < 25.0 | 1.54 | ( | 0.86 | - | 2.78 | ) | 0.149 | 1.80 | ( | 1.68 | - | 1.92 | ) | <0.001 |
|  | 25.0 ≤ BMI < 30.0 | 2.61 | ( | 1.42 | - | 4.79 | ) | 0.002 | 2.94 | ( | 2.74 | - | 3.15 | ) | <0.001 |
|  | ≥30.0 | 3.64 | ( | 1.84 | - | 7.21 | ) | <0.001 | 4.21 | ( | 3.89 | - | 4.56 | ) | <0.001 |
| Family history of hypertension | Negative | Ref | ( |  | - |  | ) |  | Ref | ( |  | - |  | ) |  |
|  | Positive | 1.34 | ( | 1.08 | - | 1.67 | ) | 0.009 | 1.13 | ( | 1.10 | - | 1.16 | ) | <0.001 |
| Cardiovascular disease | Negative | Ref | ( |  | - |  | ) |  | Ref | ( |  | - |  | ) |  |
|  | Positive | 0.88 | ( | 0.29 | - | 2.65 | ) | 0.820 | 0.65 | ( | 0.55 | - | 0.76 | ) | <0.001 |
| Considering Suicide | Negative | Ref | ( |  | - |  | ) |  | Ref | ( |  | - |  | ) |  |
|  | Positive | 0.79 | ( | 0.38 | - | 1.66 | ) | 0.532 | 1.24 | ( | 1.14 | - | 1.35 | ) | <0.001 |
| 1) Adjusted with all explanatory variables | | | | | | | | | | | | | | | |
| 2) Weight adjusted and adjusted with all explanatory variables | | | | | | | | | | | | | | | |
