# Supplementary material for: Gender with marital status, cultural differences, and vulnerability to hypertension: Findings from the national survey for noncommunicable disease risk factors and mental health using WHO STEPS in Bhutan
Source: PLoS One. 2021 Aug 31;16(8):e0256811. doi: 10.1371/journal.pone.0256811 (PMC8407566; doi:10.1371/journal.pone.0256811)
Supplement: S3 Table — (DOCX) [file pone.0256811.s007.docx]

**S3 Sensitivity analysis**

**S3 Table.** **Multivariable Logistic Regression Analysis for Hypertension with Sociocultural, Lifestyle, Biomedical, and Mental Health Variables: High blood pressure was defined as being diagnosed or under medication for hypertension, or a mean SBP ≥180 mmHg or DBP≥120 mmHg, and SBP≧160 mmHg or DBP≧100 mmHg, according to the severity of hypertension categories**

|  |  | 160 mmHg /100 mmHg^3)^ | | | | | | | | | | | | 180 mmHg/120 mmHg^4)^ | | | | | | | | | | | |
| --- | --- | --- | --- | --- | --- | --- | --- | --- | --- | --- | --- | --- | --- | --- | --- | --- | --- | --- | --- | --- | --- | --- | --- | --- | --- |
|  |  | Non-weighted | | | | | | Weighted | | | | | | Non-weighted | | | | | | Weighted | | | | | |
| n=1909 |  | AOR^1)^ | 95%CI | | | | | AOR^2)^ | 95%CI | | | | | AOR^1)^ | 95%CI | | | | | AOR^2)^ | 95%CI | | | | |
| Gender-Marital Status | Men-Married or cohabitant | Ref | ( |  | - |  | ) | Ref | ( |  | - |  | ) | Ref | ( |  | - |  | ) | Ref | ( |  | - |  | ) |
|  | Men-Never married | 0.96 | ( | 0.42 | - | 2.21 | ) | 0.73 | ( | 0.67 | - | 0.79 | ) | 0.77 | ( | 0.28 | - | 2.11 | ) | 0.42 | ( | 0.38 | - | 0.48 | ) |
|  | Men-Separated or Divorced or Widow | 1.32 | ( | 0.64 | - | 2.74 | ) | 1.32 | ( | 1.21 | - | 1.44 | ) | 1.62 | ( | 0.74 | - | 3.55 | ) | 1.82 | ( | 1.66 | - | 2.00 | ) |
|  | Women-Married or cohabitant | 1.54 | ( | 1.16 | - | 2.05 | ) | 1.58 | ( | 1.53 | - | 1.63 | ) | 1.52 | ( | 1.11 | - | 2.07 | ) | 1.70 | ( | 1.64 | - | 1.77 | ) |
|  | Women-Never married | 0.23 | ( | 0.05 | - | 0.98 | ) | 0.06 | ( | 0.04 | - | 0.08 | ) | 0.00 | ( | 0.00 | - | . | ) | 0.00 | ( | 0.00 | - | . | ) |
|  | Women-Separated or Divorced or Widow | 1.75 | ( | 1.18 | - | 2.59 | ) | 1.46 | ( | 1.37 | - | 1.54 | ) | 1.72 | ( | 1.13 | - | 2.63 | ) | 1.54 | ( | 1.45 | - | 1.64 | ) |
| Age | 18-29 years | Ref | ( |  | - |  | ) | Ref | ( |  | - |  | ) | Ref | ( |  | - |  | ) | Ref | ( |  | - |  | ) |
|  | 30-39 years | 2.06 | ( | 1.39 | - | 3.08 | ) | 2.42 | ( | 2.32 | - | 2.53 | ) | 1.86 | ( | 1.20 | - | 2.90 | ) | 2.13 | ( | 2.02 | - | 2.24 | ) |
|  | 40-49 years | 3.69 | ( | 2.45 | - | 5.56 | ) | 3.86 | ( | 3.67 | - | 4.05 | ) | 3.17 | ( | 2.02 | - | 4.97 | ) | 3.91 | ( | 3.70 | - | 4.13 | ) |
|  | 50-59 years | 5.49 | ( | 3.52 | - | 8.56 | ) | 6.57 | ( | 6.23 | - | 6.92 | ) | 4.78 | ( | 2.94 | - | 7.75 | ) | 5.91 | ( | 5.57 | - | 6.27 | ) |
|  | 60-69 years | 7.72 | ( | 4.67 | - | 12.75 | ) | 9.75 | ( | 9.18 | - | 10.35 | ) | 5.86 | ( | 3.41 | - | 10.05 | ) | 8.82 | ( | 8.25 | - | 9.43 | ) |
| Education-years | No formal education | Ref | ( |  | - |  | ) | Ref | ( |  | - |  | ) | Ref | ( |  | - |  | ) | Ref | ( |  | - |  | ) |
|  | 1-10 years | 0.97 | ( | 0.73 | - | 1.29 | ) | 1.07 | ( | 1.04 | - | 1.11 | ) | 0.97 | ( | 0.71 | - | 1.33 | ) | 1.02 | ( | 0.98 | - | 1.05 | ) |
|  | 11-12 years | 0.79 | ( | 0.34 | - | 1.84 | ) | 1.11 | ( | 1.02 | - | 1.20 | ) | 0.64 | ( | 0.23 | - | 1.78 | ) | 0.87 | ( | 0.78 | - | 0.96 | ) |
|  | More than 12 years | 1.08 | ( | 0.46 | - | 2.55 | ) | 0.77 | ( | 0.69 | - | 0.86 | ) | 1.17 | ( | 0.46 | - | 2.97 | ) | 1.06 | ( | 0.94 | - | 1.18 | ) |
| Working Status | Employee | Ref | ( |  | - |  | ) | Ref | ( |  | - |  | ) | Ref | ( |  | - |  | ) | Ref | ( |  | - |  | ) |
|  | Self-employed | 0.88 | ( | 0.60 | - | 1.29 | ) | 1.12 | ( | 1.08 | - | 1.17 | ) | 0.90 | ( | 0.59 | - | 1.38 | ) | 0.97 | ( | 0.92 | - | 1.01 | ) |
|  | Non-working | 0.92 | ( | 0.61 | - | 1.38 | ) | 1.10 | ( | 1.05 | - | 1.16 | ) | 1.03 | ( | 0.66 | - | 1.60 | ) | 1.18 | ( | 1.12 | - | 1.25 | ) |
| Residential area | Rural | Ref | ( |  | - |  | ) | Ref | ( |  | - |  | ) | Ref | ( |  | - |  | ) | Ref | ( |  | - |  | ) |
|  | Urban | 0.95 | ( | 0.71 | - | 1.28 | ) | 0.92 | ( | 0.89 | - | 0.96 | ) | 1.13 | ( | 0.82 | - | 1.56 | ) | 1.14 | ( | 1.10 | - | 1.19 | ) |
| Income | Nu.0-9,000 | Ref | ( |  | - |  | ) | Ref | ( |  | - |  | ) | Ref | ( |  | - |  | ) | Ref | ( |  | - |  | ) |
|  | Nu.9,001-30,000 | 1.38 | ( | 1.03 | - | 1.87 | ) | 1.33 | ( | 1.28 | - | 1.37 | ) | 1.26 | ( | 0.91 | - | 1.74 | ) | 1.11 | ( | 1.07 | - | 1.15 | ) |
|  | Nu.30,001-60,000 | 1.32 | ( | 0.93 | - | 1.89 | ) | 1.27 | ( | 1.21 | - | 1.32 | ) | 1.18 | ( | 0.81 | - | 1.74 | ) | 1.20 | ( | 1.15 | - | 1.26 | ) |
|  | Nu.60,001- | 1.11 | ( | 0.76 | - | 1.61 | ) | 1.18 | ( | 1.13 | - | 1.24 | ) | 0.84 | ( | 0.56 | - | 1.27 | ) | 0.79 | ( | 0.75 | - | 0.84 | ) |
| Survey language | Dzongkha | Ref | ( |  | - |  | ) | Ref | ( |  | - |  | ) | Ref | ( |  | - |  | ) | Ref | ( |  | - |  | ) |
|  | Tshanglakha | 1.60 | ( | 1.20 | - | 2.15 | ) | 1.26 | ( | 1.22 | - | 1.31 | ) | 1.29 | ( | 0.94 | - | 1.78 | ) | 1.06 | ( | 1.02 | - | 1.11 | ) |
|  | Lhotshamkha | 1.61 | ( | 1.19 | - | 2.17 | ) | 1.21 | ( | 1.17 | - | 1.26 | ) | 1.47 | ( | 1.06 | - | 2.03 | ) | 1.08 | ( | 1.04 | - | 1.12 | ) |
|  | English | 0.38 | ( | 0.08 | - | 1.78 | ) | 0.17 | ( | 0.14 | - | 0.21 | ) | 0.55 | ( | 0.12 | - | 2.58 | ) | 0.24 | ( | 0.19 | - | 0.29 | ) |
| Tobacco use | Never use | Ref | ( |  | - |  | ) | Ref | ( |  | - |  | ) | Ref | ( |  | - |  | ) | Ref | ( |  | - |  | ) |
|  | Currently use | 0.61 | ( | 0.44 | - | 0.84 | ) | 0.69 | ( | 0.67 | - | 0.72 | ) | 0.57 | ( | 0.40 | - | 0.81 | ) | 0.67 | ( | 0.65 | - | 0.70 | ) |
| Alcohol consumption | Never drink | Ref | ( |  | - |  | ) | Ref | ( |  | - |  | ) | Ref | ( |  | - |  | ) | Ref | ( |  | - |  | ) |
|  | Light or moderate drinking | 1.08 | ( | 0.83 | - | 1.42 | ) | 1.02 | ( | 0.99 | - | 1.05 | ) | 0.89 | ( | 0.66 | - | 1.20 | ) | 0.88 | ( | 0.84 | - | 0.91 | ) |
|  | Heavy drinking | 1.75 | ( | 1.30 | - | 2.34 | ) | 1.48 | ( | 1.43 | - | 1.53 | ) | 1.39 | ( | 1.02 | - | 1.91 | ) | 1.37 | ( | 1.32 | - | 1.43 | ) |
| Fruit and vegetable consumption | more than 5 serves per day | Ref | ( |  | - |  | ) | Ref | ( |  | - |  | ) | Ref | ( |  | - |  | ) | Ref | ( |  | - |  | ) |
|  | 5 or fewer serves per day | 1.30 | ( | 1.01 | - | 1.68 | ) | 1.19 | ( | 1.15 | - | 1.22 | ) | 1.42 | ( | 1.07 | - | 1.87 | ) | 1.49 | ( | 1.44 | - | 1.54 | ) |
| Physical Activity | 150 min or more per week | Ref | ( |  | - |  | ) | Ref | ( |  | - |  | ) | Ref | ( |  | - |  | ) | Ref | ( |  | - |  | ) |
|  | Less than 150 min per week | 0.74 | ( | 0.48 | - | 1.14 | ) | 0.68 | ( | 0.64 | - | 0.72 | ) | 0.71 | ( | 0.45 | - | 1.14 | ) | 0.63 | ( | 0.59 | - | 0.67 | ) |
| Salt intake | Less than 5 g per day | Ref | ( |  | - |  | ) | Ref | ( |  | - |  | ) | Ref | ( |  | - |  | ) | Ref | ( |  | - |  | ) |
|  | 5 g or more per day | 1.16 | ( | 0.42 | - | 3.23 | ) | 2.22 | ( | 1.88 | - | 2.62 | ) | 0.76 | ( | 0.27 | - | 2.14 | ) | 1.31 | ( | 1.10 | - | 1.55 | ) |
| Blood glucose | Normal | Ref | ( |  | - |  | ) | Ref | ( |  | - |  | ) | Ref | ( |  | - |  | ) | Ref | ( |  | - |  | ) |
|  | Abnormal | 1.55 | ( | 1.06 | - | 2.27 | ) | 1.59 | ( | 1.51 | - | 1.66 | ) | 1.47 | ( | 1.00 | - | 2.18 | ) | 1.55 | ( | 1.47 | - | 1.63 | ) |
| Total cholesterol | Less than 240 mg/dl | Ref | ( |  | - |  | ) | Ref | ( |  | - |  | ) | Ref | ( |  | - |  | ) | Ref | ( |  | - |  | ) |
|  | 240 mg/dl or more | 1.57 | ( | 0.77 | - | 3.20 | ) | 1.77 | ( | 1.62 | - | 1.93 | ) | 1.62 | ( | 0.78 | - | 3.36 | ) | 2.35 | ( | 2.14 | - | 2.57 | ) |
| BMI | <18.5 | Ref | ( |  | - |  | ) | Ref | ( |  | - |  | ) | Ref | ( |  | - |  | ) | Ref | ( |  | - |  | ) |
|  | 18.5 ≤ BMI < 25.0 | 1.11 | ( | 0.57 | - | 2.17 | ) | 1.14 | ( | 1.05 | - | 1.23 | ) | 1.15 | ( | 0.54 | - | 2.45 | ) | 1.03 | ( | 0.94 | - | 1.12 | ) |
|  | 25.0 ≤ BMI < 30.0 | 2.05 | ( | 1.03 | - | 4.05 | ) | 1.95 | ( | 1.80 | - | 2.11 | ) | 2.51 | ( | 1.16 | - | 5.45 | ) | 2.11 | ( | 1.93 | - | 2.30 | ) |
|  | ≥30.0 | 2.38 | ( | 1.13 | - | 5.02 | ) | 2.98 | ( | 2.73 | - | 3.25 | ) | 2.74 | ( | 1.19 | - | 6.32 | ) | 3.10 | ( | 2.81 | - | 3.42 | ) |
| Family history of hypertension | Negative | Ref | ( |  | - |  | ) | Ref | ( |  | - |  | ) | Ref | ( |  | - |  | ) | Ref | ( |  | - |  | ) |
|  | Positive | 1.50 | ( | 1.18 | - | 1.90 | ) | 1.47 | ( | 1.43 | - | 1.51 | ) | 1.78 | ( | 1.38 | - | 2.30 | ) | 1.85 | ( | 1.80 | - | 1.91 | ) |
| Cardiovascular disease | Negative | Ref | ( |  | - |  | ) | Ref | ( |  | - |  | ) | Ref | ( |  | - |  | ) | Ref | ( |  | - |  | ) |
|  | Positive | 1.12 | ( | 0.36 | - | 3.47 | ) | 0.91 | ( | 0.77 | - | 1.06 | ) | 1.42 | ( | 0.46 | - | 4.36 | ) | 1.16 | ( | 0.99 | - | 1.36 | ) |
| Considering Suicide | Negative | Ref | ( |  | - |  | ) | Ref | ( |  | - |  | ) | Ref | ( |  | - |  | ) | Ref | ( |  | - |  | ) |
|  | Positive | 0.72 | ( | 0.30 | - | 1.73 | ) | 0.74 | ( | 0.67 | - | 0.83 | ) | 0.87 | ( | 0.35 | - | 2.18 | ) | 0.97 | ( | 0.86 | - | 1.09 | ) |
| 1) Adjusted with all explanatory variables | |  |  |  |  |  |  |  |  |  |  |  |  |  |  |  |  |  |  |  |  |  |  |  |  |
| 2) Weight adjusted and adjusted with all explanatory variables | | | | | | | | | | | | | | | | | | | |  |  |  |  |  |  |
| 3) High blood pressure: defined as either a mean systolic blood pressure (SBP) ≥160 mmHg or a mean diastolic blood pressure (DBP) ≥100 mmHg or previously diagnosed by healthcare workers or currently under medication for raised blood pressure | | | | | | | | | | | | | | | | | | | | | | | | | |
| 4) High blood pressure: defined as either a mean systolic blood pressure (SBP) ≥180 mmHg or a mean diastolic blood pressure (DBP) ≥120 mmHg or previously diagnosed by healthcare workers or currently under medication for raised blood pressure | | | | | | | | | | | | | | | | | | | | | | | | | |
