# Supplementary material for: Gender with marital status, cultural differences, and vulnerability to hypertension: Findings from the national survey for noncommunicable disease risk factors and mental health using WHO STEPS in Bhutan
Source: PLoS One. 2021 Aug 31;16(8):e0256811. doi: 10.1371/journal.pone.0256811 (PMC8407566; doi:10.1371/journal.pone.0256811)
Supplement: S4 Table — (DOCX) [file pone.0256811.s008.docx]

**S4 Multiple imputation analysis**

**S4 Table. Multivariable Logistic Regression Analysis for Hypertension with Sociocultural, Lifestyle, Biomedical, and Mental Health Variables: Multiple imputation**

|  |  | Non-weighted | | | | | | | Weighted | | | | | | | |
| --- | --- | --- | --- | --- | --- | --- | --- | --- | --- | --- | --- | --- | --- | --- | --- | --- |
|  |  | AOR^1)^ | 95%CI | | | | | *p*-value | AOR^2)^ | 95%CI | | | | | *p-*value | |
| Gender-Marital Status | Men-Married or cohabitant | Ref | ( |  | - |  | ) |  | Ref | ( |  | - |  | ) | |  |
|  | Men-Never married | 1.79 | ( | 1.03 | - | 3.10 | ) | 0.038 | 1.49 | ( | 1.41 | - | 1.59 | ) | | <0.001 |
|  | Men-Separated or Divorced or Widow | 0.66 | ( | 0.35 | - | 1.24 | ) | 0.197 | 0.62 | ( | 0.57 | - | 0.67 | ) | | <0.001 |
|  | Women-Married or cohabitant | 1.16 | ( | 0.92 | - | 1.47 | ) | 0.217 | 1.14 | ( | 1.11 | - | 1.17 | ) | | <0.001 |
|  | Women-Never married | 0.65 | ( | 0.36 | - | 1.20 | ) | 0.168 | 0.59 | ( | 0.54 | - | 0.65 | ) | | <0.001 |
|  | Women-Separated or Divorced or Widow | 1.26 | ( | 0.89 | - | 1.79 | ) | 0.196 | 1.28 | ( | 1.21 | - | 1.35 | ) | | <0.001 |
| Age | 18-29 years | Ref | ( |  | - |  | ) |  | Ref | ( |  | - |  | ) | |  |
|  | 30-39 years | 1.96 | ( | 1.46 | - | 2.63 | ) | <0.001 | 2.33 | ( | 2.26 | - | 2.41 | ) | | <0.001 |
|  | 40-49 years | 3.53 | ( | 2.58 | - | 4.82 | ) | <0.001 | 3.38 | ( | 3.26 | - | 3.51 | ) | | <0.001 |
|  | 50-59 years | 4.64 | ( | 3.28 | - | 6.58 | ) | <0.001 | 4.68 | ( | 4.48 | - | 4.88 | ) | | <0.001 |
|  | 60-69 years | 6.67 | ( | 4.40 | - | 10.09 | ) | <0.001 | 6.91 | ( | 6.56 | - | 7.27 | ) | | <0.001 |
| Education-years | No formal education | Ref | ( |  | - |  | ) |  | Ref | ( |  | - |  | ) | |  |
|  | 1-10 years | 0.95 | ( | 0.75 | - | 1.21 | ) | 0.674 | 0.96 | ( | 0.94 | - | 0.99 | ) | | 0.006 |
|  | 11-12 years | 0.55 | ( | 0.30 | - | 1.00 | ) | 0.051 | 0.61 | ( | 0.57 | - | 0.65 | ) | | <0.001 |
|  | More than 12 years | 1.31 | ( | 0.68 | - | 2.52 | ) | 0.413 | 1.17 | ( | 1.08 | - | 1.27 | ) | | <0.001 |
| Working Status | Employee | Ref | ( |  | - |  | ) |  | Ref | ( |  | - |  | ) | |  |
|  | Self-employed | 1.00 | ( | 0.73 | - | 1.37 | ) | 0.997 | 1.16 | ( | 1.11 | - | 1.20 | ) | | <0.001 |
|  | Non-working | 0.88 | ( | 0.63 | - | 1.24 | ) | 0.470 | 1.02 | ( | 0.98 | - | 1.06 | ) | | 0.433 |
| Residential area | Rural | Ref | ( |  | - |  | ) |  | Ref | ( |  | - |  | ) | |  |
|  | Urban | 1.04 | ( | 0.80 | - | 1.34 | ) | 0.784 | 1.19 | ( | 1.15 | - | 1.23 | ) | | <0.001 |
| Income | Nu.0-9,000 | Ref | ( |  | - |  | ) |  | Ref | ( |  | - |  | ) | |  |
|  | Nu.9,001-30,000 | 1.13 | ( | 0.88 | - | 1.46 | ) | 0.330 | 0.91 | ( | 0.85 | - | 0.98 | ) | | 0.010 |
|  | Nu.30,001-60,000 | 1.04 | ( | 0.76 | - | 1.41 | ) | 0.825 | 0.73 | ( | 0.67 | - | 0.78 | ) | | <0.001 |
|  | Nu.60,001- | 1.12 | ( | 0.82 | - | 1.53 | ) | 0.467 | 1.04 | ( | 0.99 | - | 1.10 | ) | | 0.156 |
| Survey language | Dzongkha | Ref | ( |  | - |  | ) |  | Ref | ( |  | - |  | ) | |  |
|  | Tshanglakha | 1.61 | ( | 1.26 | - | 2.06 | ) | <0.001 | 1.15 | ( | 1.11 | - | 1.20 | ) | | <0.001 |
|  | Lhotshamkha | 1.30 | ( | 1.02 | - | 1.67 | ) | 0.035 | 1.01 | ( | 0.98 | - | 1.04 | ) | | 0.729 |
|  | English | 0.42 | ( | 0.16 | - | 1.09 | ) | 0.075 | 0.40 | ( | 0.36 | - | 0.44 | ) | | <0.001 |
| Tobacco use | Never use | Ref | ( |  | - |  | ) |  | Ref | ( |  | - |  | ) | |  |
|  | Currently use | 0.76 | ( | 0.59 | - | 0.97 | ) | 0.030 | 0.84 | ( | 0.81 | - | 0.86 | ) | | <0.001 |
| Alcohol consumption | Never drink | Ref | ( |  | - |  | ) |  | Ref | ( |  | - |  | ) | |  |
|  | Light or moderate drinking | 1.21 | ( | 0.97 | - | 1.52 | ) | 0.097 | 1.14 | ( | 1.11 | - | 1.17 | ) | | <0.001 |
|  | Heavy drinking | 1.55 | ( | 1.20 | - | 1.99 | ) | 0.001 | 1.24 | ( | 1.20 | - | 1.28 | ) | | <0.001 |
| Fruit and vegetable consumption | More than 5 serves per day | Ref | ( |  | - |  | ) |  | Ref | ( |  | - |  | ) | |  |
|  | 5 for fewer serves per day | 1.09 | ( | 0.88 | - | 1.37 | ) | 0.435 | 0.99 | ( | 0.91 | - | 1.06 | ) | | 0.691 |
| Physical Activity | 150 min or more per week | Ref | ( |  | - |  | ) |  | Ref | ( |  | - |  | ) | |  |
|  | Less than 150 min per week | 0.77 | ( | 0.53 | - | 1.12 | ) | 0.170 | 0.79 | ( | 0.75 | - | 0.83 | ) | | <0.001 |
| Salt intake | Less than 5 g per day | Ref | ( |  | - |  | ) |  | Ref | ( |  | - |  | ) | |  |
|  | 5 g or more per day | 1.38 | ( | 0.57 | - | 3.33 | ) | 0.478 | 1.49 | ( | 0.84 | - | 2.65 | ) | | 0.158 |
| Blood glucose | Normal | Ref | ( |  | - |  | ) |  | Ref | ( |  | - |  | ) | |  |
|  | Abnormal | 1.98 | ( | 1.37 | - | 2.86 | ) | <0.001 | 2.08 | ( | 1.88 | - | 2.31 | ) | | <0.001 |
| Total cholesterol | Less than 240 mg/dl | Ref | ( |  | - |  | ) |  | Ref | ( |  | - |  | ) | |  |
|  | 240 mg/dl or more | 1.49 | ( | 0.75 | - | 2.93 | ) | 0.253 | 1.83 | ( | 1.35 | - | 2.48 | ) | | <0.001 |
| BMI | <18.5 | Ref | ( |  | - |  | ) |  | Ref | ( |  | - |  | ) | |  |
|  | 18.5 ≤ BMI < 25.0 | 1.47 | ( | 0.87 | - | 2.49 | ) | 0.149 | 1.69 | ( | 1.58 | - | 1.81 | ) | | <0.001 |
|  | 25.0 ≤ BMI < 30.0 | 2.51 | ( | 1.46 | - | 4.32 | ) | 0.001 | 2.85 | ( | 2.66 | - | 3.05 | ) | | <0.001 |
|  | ≥30.0 | 3.55 | ( | 1.90 | - | 6.62 | ) | <0.001 | 4.09 | ( | 3.79 | - | 4.42 | ) | | <0.001 |
| Family history of hypertension | Negative | Ref | ( |  | - |  | ) |  | Ref | ( |  | - |  | ) | |  |
|  | Positive | 1.32 | ( | 1.07 | - | 1.62 | ) | 0.008 | 1.12 | ( | 1.09 | - | 1.14 | ) | | <0.001 |
| Cardiovascular disease | Negative | Ref | ( |  | - |  | ) |  | Ref | ( |  | - |  | ) | |  |
|  | Positive | 0.96 | ( | 0.33 | - | 2.77 | ) | 0.938 | 0.71 | ( | 0.61 | - | 0.82 | ) | | <0.001 |
| Considering Suicide | Negative | Ref | ( |  | - |  | ) |  | Ref | ( |  | - |  | ) | |  |
|  | Positive | 0.90 | ( | 0.45 | - | 1.79 | ) | 0.756 | 1.08 | ( | 0.98 | - | 1.18 | ) | | 0.104 |
| 1) Adjusted with all explanatory variables | | | | | | | | | | | | | | | | |
| 2) Weight adjusted and adjusted with all explanatory variables | | | | | | | | | | | | | | | | |
| The missing value analysis did not show any obvious bias. However, we would like to show the results of multiple imputation. The number of imputations was 20 times, including all variables which we used in the multivariable logistic regression models. The multiple imputation method was regression-based imputation, using IBM SPSS Statistics version 23 (IBM Corp., Armonk, NY, USA). | | | | | | | | | | | | | | | | |
