# Supplementary material for: Gender with marital status, cultural differences, and vulnerability to hypertension: Findings from the national survey for noncommunicable disease risk factors and mental health using WHO STEPS in Bhutan
Source: PLoS One. 2021 Aug 31;16(8):e0256811. doi: 10.1371/journal.pone.0256811 (PMC8407566; doi:10.1371/journal.pone.0256811)
Supplement: S5 Table — Estimated Salt Intake per day(g) by Survey language. (DOCX) [file pone.0256811.s009.docx]

**S5 Estimated salt intake by survey language category**

**S5 Table. Estimated Salt Intake per day(g) by Survey language**

|  | N | Mean | SD |
| --- | --- | --- | --- |
| Dzongkha | 679 | 13.09 | 9.911 |
| Tshanglakha | 667 | 16.98 | 17.154 |
| Lhotshamkha | 531 | 13.23 | 9.715 |
| English | 32 | 12.34 | 5.154 |
| Total | 1909 | 14.47 | 12.948 |
